# Pediatric Residents’ Perceptions of a Point-of-Care Ultrasound Collaboration With Emergency Medicine

**DOI:** 10.7759/cureus.41645

**Published:** 2023-07-10

**Authors:** Brandon M Wubben, Megan Oberbillig, Cory Wittrock, Kacie Rytlewski, Caitlin K Thirnbeck, Christian Junker, Amy Stier

**Affiliations:** 1 Emergency Medicine, University of Iowa, Iowa City, USA; 2 Pediatrics, University of Iowa, Iowa City, USA

**Keywords:** point-of-care ultrasonography, pocus, ultrasound education, medical education, emergency medicine ultrasound, pediatrics residents

## Abstract

Background

Pediatric residencies expanding their point-of-care ultrasound (POCUS) education face barriers, including a lack of established curriculum and qualified educators. Prior studies report partnerships between pediatrics and pediatric emergency medicine (PEM); however, many non-PEM emergency medicine (EM) physicians with POCUS fellowship training also have experience with pediatric POCUS and represent an alternate educational partner.

Objectives

To improve pediatric residents' POCUS skills through collaborative education with EM and evaluate perceptions of the teaching format and instructors.

Methods

First through third-year pediatric residents attended a half-day didactic and hands-on session about renal, lung, and musculoskeletal (MSK) POCUS. These educational sessions were led by EM faculty with POCUS fellowship training and assisted by EM residents. Post-session surveys were administered to pediatric residents to assess prior POCUS experience, changes in confidence in acquiring and interpreting renal, lung, and MSK POCUS images, and opinions about the educational format. Statistical analyses of the post-session survey data were performed using SPSS.

Results

Thirty-nine pediatric residents attended the session and completed the survey of 45 total residents in the program (86.7%), with 89.7% completing 10 or fewer POCUS studies. Residents' comfort level with performing lung POCUS increased from 5.1% to 82.1% (*p *< .001), renal POCUS from 10.3% to 76.9% (*p *< .001), and MSK POCUS from 7.7% to 84.6% (*p *< .001). 87.2% rated the educational format as effective, and 94.9% (37/39) rated emergency medicine faculty as 'very effective' in providing ultrasound education relevant to the practice of pediatrics.

Conclusion

Pediatric resident POCUS education taught by EM faculty with POCUS fellowship training was well-received by pediatric residents and significantly improved confidence in acquiring and interpreting POCUS.

## Introduction

Multiple specialties have increasingly utilized point-of-care ultrasound (POCUS), including emergency medicine, obstetrics, internal medicine, and subspecialties such as neonatology, pediatric emergency medicine, and pediatric critical care [1-4]. POCUS provides benefits such as faster diagnosis and treatment without ionizing radiation and can be used for a wide range of applications, including procedural guidance, thoracic, renal, cardiac, soft tissue, musculoskeletal, and bowel exams [5,6].

When surveyed, most pediatric residents believed their residency programs should teach them how to use POCUS [7]. However, many pediatric residencies do not currently teach POCUS, with reported barriers including a lack of an established curriculum and a lack of qualified educators [7-9]. Some residency programs have begun to address these barriers, including one program that implemented a three-year longitudinal curriculum designed by pediatric emergency medicine (PEM) and POCUS fellowship-trained provider [10]. This provides a natural partnership for pediatric residency programs with a PEM training pathway. However, many non-PEM emergency medicine providers also have substantial experience performing POCUS on pediatric patients, as formal POCUS education has been a component of emergency medicine residency education for over a decade [4]. The American Board of Emergency Medicine also offers a POCUS-Focused Practice Designation after completing an accredited POCUS fellowship program, encompassing adult and pediatric POCUS applications [4].

In addition to pediatric faculty with POCUS experience and PEM providers, we propose that partnerships between pediatrics and POCUS fellowship-trained emergency medicine providers may represent an additional source of qualified educators for POCUS basics. The goal of this study was to evaluate the effect of a pilot pediatric POCUS curriculum taught by POCUS fellowship-trained emergency medicine providers on pediatric residents' comfort levels with performing POCUS and to assess residents' perceptions of the teaching format, instructors, and likelihood of using POCUS in their clinical practice.

## Materials and methods

Our pediatric residency program holds half-day educational sessions on focused topics throughout the year as part of regularly scheduled resident education. There were 45 residents in the pediatric residency program at the time of the POCUS session; not all residents could attend due to a portion of residents being on night rotations and vacation. The session (conducted in Spring 2023) consisted of a 30-minute introductory lecture given by a POCUS fellowship-trained emergency medicine faculty member about the basics of POCUS. The content included basic ultrasound physics, scanning techniques, and a review of recommendations on POCUS use that pediatric experts have published. This introduction was followed by three presentations lasting 10 minutes on the basics of thoracic, renal, and musculoskeletal (MSK) POCUS exams. Residents were then divided into small groups for hands-on teaching and practice with an emergency medicine faculty instructor. Emergency medicine residents assisted in teaching and served as ultrasound models, as well as some pediatric residents that volunteered to serve as ultrasound models for their peers, resulting in an instructor-to-participant ratio ranging between 1:6 and 1:12. Ultrasound platforms used for hands-on practice included the portable handheld system Butterfly iQ+ (Butterfly Network, Burlington, MA), and the cart-based systems Sonosite PX (Fujifilm Inc., Bothell, WA) and Philips Sparq (Philips, Bothell, WA).

Post-session surveys were distributed to pediatric residents to record the level of training, prior experience, perceived changes in POCUS comfort level, perceived likelihood of POCUS use in clinical practice, and opinions about the teaching format and suitability of instructors. Descriptive statistics were analyzed. Before and after paired categorical survey responses were compared using the X2 McNemar test, with 'very comfortable' and 'somewhat comfortable' binned as 'comfortable' and 'very uncomfortable' and 'somewhat uncomfortable' binned as 'uncomfortable' for dichotomous analysis. SPSS (IBM SPSS Statistics for Macintosh, Version 28.0, Armonk, NY) was used for statistical analysis. This study was granted a waiver of written informed consent by our institutional review board; eligible participants were informed verbally and with a printed handout regarding the study procedures, and residents choosing to participate filled out an anonymous paper survey.

## Results

**Figure 1:**
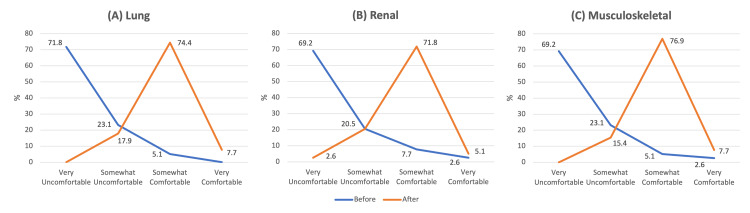
Pediatric residents’ self-reported confidence in acquiring and interpreting ultrasound images for lung (A), renal (B) and musculoskeletal (C) point-of-care ultrasound applications before and after the educational session.

Thirty-nine pediatric residents attended the session and completed the survey of 45 total residents in the program (86.7%). Prior experience with POCUS was low, with 89.7% (35/39) having completed 10 or fewer ultrasounds, of whom 30.8% (12/39) had completed zero ultrasounds previously. Most residents were very uncomfortable with lung, renal, and MSK POCUS before the educational session (Figures 1A-1C). The percentage of residents comfortable with lung POCUS increased from 5.1% to 82.1% (p < .001), renal POCUS from 10.3% to 76.9% (p < .001), and MSK POCUS from 7.7% to 84.6% (p < .001). About half of residents reported they were very or somewhat likely to use lung POCUS (46.2%) and MSK POCUS (48.7%) in their practice, compared to 33.3% of residents likely to use renal POCUS.

All residents reported that the ultrasound education was useful, with 87.2% rating the educational format as effective. The pediatric residents' ratings of emergency medicine faculty as instructors were favorable, with 94.9% (37/39) selecting that they were 'very effective' in providing ultrasound education relevant to the practice of pediatrics. One resident reported the instructors were 'somewhat effective', and one reported they were 'somewhat ineffective'.

## Discussion

We found that a half-day didactic and hands-on session about the basics of renal, lung, and MSK POCUS taught by EM faculty with POCUS fellowship training was well-received by pediatric residents and had a large impact on their self-reported confidence in acquiring and interpreting these types of POCUS studies. Baseline POCUS experience was very low among our pediatric residents, with 89.7% having performed 10 or fewer ultrasounds previously, similar to the baseline experience for a previously reported pilot curriculum taught by PEM providers (84%) [11]. The large increases in application-specific pediatric resident confidence with POCUS in our study of 66-77% were similar to those in a longer longitudinal curriculum (61-90%) [10].

The current study suggests that partnerships between pediatrics and emergency medicine may be an effective strategy for teaching POCUS basics to pediatric residents because a lack of qualified educators within pediatrics is reported as a barrier to providing this education [7-9]. While other programs have effectively utilized PEM program faculty to teach POCUS, many pediatric residency programs do not have access to a PEM training program [10,11]. Our institution's emergency medicine ultrasound faculty already provide similar training on POCUS basics to medical students, new emergency medicine interns, and application-specific education to residents in other specialties such as family medicine, neurology, and general surgery. In our experience, many emergency medicine ultrasound programs already have the infrastructure and equipment to provide this education.

The majority of residents reported moving from "very uncomfortable" to "somewhat comfortable" for the three POCUS types taught in this session; however, the number of residents who were "very comfortable" was still very low. While EM faculty may be effective at teaching POCUS basics, without pediatric faculty who are experienced in performing and teaching POCUS in the clinical setting, pediatric residents may be unlikely to succeed at further improving their POCUS confidence. The current study does provide evidence that utilizing emergency medicine faculty to teach pediatric residents basic POCUS skills was well received, which can inform the development of more longitudinal curricula by helping to address the reported shortage of POCUS educators within pediatrics. We hope pediatric residents can build on the basic ultrasound skills from this session when working clinically with pediatric subspecialists with POCUS experience. However, more research is needed to determine if this approach is effective. Future studies could also incorporate self-directed pre-session online education and longitudinal assessments, including quantitative comparisons using pre and post-session testing of objective knowledge of the material covered in the didactics and hands-on sessions.

Limitations

This was a single-site study, and experience may vary widely among academic centers, including the amount of baseline POCUS experience among pediatric residents and the amount of pediatric experience among emergency medicine faculty. However, the baseline POCUS experience of our residents was similar to the previously published baseline experience, and the minimum content for successfully completing a POCUS fellowship is relatively standardized [12]. Another limitation of our study is that residents may have answered questions more favorably due to the power differential between learners and instructors; we attempted to minimize this by emphasizing that participation in the survey was completely voluntary and anonymous. The survey instrument was based on prior pediatric POCUS education research but was not independently validated. Feedback from instructors was also not obtained, which may have strengthened the overall assessment. In addition, the current study was not designed to assess improvements in clinical POCUS proficiency but rather to assess a subcomponent of a more comprehensive education plan. Further study is needed to determine whether EM educators can successfully be integrated into a longitudinal pediatric POCUS curriculum.

## Conclusions

Many pediatric residents have minimal baseline POCUS experience, and before this educational intervention, most reported being very uncomfortable with using POCUS. We found that a didactic and hands-on educational collaboration between our pediatrics residency and emergency medicine faculty significantly improved self-reported confidence in acquiring and interpreting POCUS studies, and many pediatric residents reported they were likely to use these skills in their practice. The collaboration was well-received by pediatric residents, who rated emergency medicine faculty favorably as instructors and demonstrated a promising partnership between pediatrics and emergency medicine for teaching basic POCUS skills. The current study provides evidence that utilizing emergency medicine faculty to help teach pediatric residents basic POCUS skills may be a viable strategy, which can inform the development of more longitudinal curricula by helping to address the current shortage of POCUS educators within pediatrics.

## Appendices

Post-session questionnaire

1.              What is your level of training: (PGY = postgraduate year):

a.              Peds PGY 1

b.              Peds PGY 2

c.              Peds PGY 3

d.              Peds PGY 4

e.              Peds PGY 5

f.               Peds Fellow

g.              Peds Faculty

h.              Other _________

2.              Before today, how many point-of-care ultrasound (POCUS) exams had you performed (either educationally or clinically)?

a.              0

b.              1-10

c.               11-50

d.              50+

3.              Before today, what was your comfort level in acquiring and interpreting ultrasound images for thoracic exams?

a.              Very comfortable

b.              Somewhat comfortable

c.               Somewhat uncomfortable

d.              Very uncomfortable

4.              After today, what is your comfort level in acquiring and interpreting ultrasound images for thoracic exams?

a.              Very comfortable

b.              Somewhat comfortable

c.               Somewhat uncomfortable

d.              Very uncomfortable

5.              Before today, what was your comfort level in acquiring and interpreting ultrasound images for renal exams?

a.              Very comfortable

b.              Somewhat comfortable

c.               Somewhat uncomfortable

d.              Very uncomfortable

6.              After today, what is your comfort level in acquiring and interpreting ultrasound images for renal exams?

a.              Very comfortable

b.              Somewhat comfortable

c.               Somewhat uncomfortable

d.              Very uncomfortable

7.              Before today, what was your comfort level in acquiring and interpreting ultrasound images for MSK exams?

a.              Very comfortable

b.              Somewhat comfortable

c.               Somewhat uncomfortable

d.              Very uncomfortable

8.              After today, what is your comfort level in acquiring and interpreting ultrasound images for MSK exams?

a.              Very comfortable

b.              Somewhat comfortable

c.               Somewhat uncomfortable

d.              Very uncomfortable

9.              Going forward, how likely are you to use thoracic ultrasound in your practice?

a.              Very likely

b.              Somewhat likely

c.               Somewhat unlikely

d.              Very unlikely

10.  Going forward, how likely are you to use renal ultrasound in your practice?

a.              Very likely

b.              Somewhat likely

c.               Somewhat unlikely

d.              Very unlikely

11.  Going forward, how likely are you to use MSK ultrasound in your practice?

a.              Very likely

b.              Somewhat likely

c.               Somewhat unlikely

d.              Very unlikely

12.  Did you find today’s ultrasound education useful?

a.              Yes

b.              No

13.  Did you find the teaching format to be effective?

a.              Very effective

b.              Somewhat effective

c.               Somewhat ineffective

d.              Very ineffective

14.  Did you find that the instructors (Emergency Medicine Faculty) were effective in providing ultrasound education relevant to the practice of pediatrics?

a.              Yes, very effective

b.              Yes, somewhat effective

c.               No, somewhat ineffective

d.              No, very ineffective
